# Supplementary material for: Time-resolved lineage recording reveals a pre-existing, heritable cell state underlying metastatic potential
Source: bioRxiv. 2026 Aug 12:2026.08.10.744013. Preprint. [Version 2] doi: 10.64898/2026.08.10.744013 (PMC13640744; doi:10.64898/2026.08.10.744013)
Supplement: Supplementary file 1 [file NIHPP2026.08.10.744013v2-supplement-9.pdf]

bioRxiv preprint doi: https://doi.org/10.64898/2026.08.10.744013; this version posted August 12, 2026. The copyright holder for this preprint (which was not certified by peer review) is the author/funder, who has granted bioRxiv a license to display the preprint in perpetuity. It is made available under a CC-BY 4.0 International license.

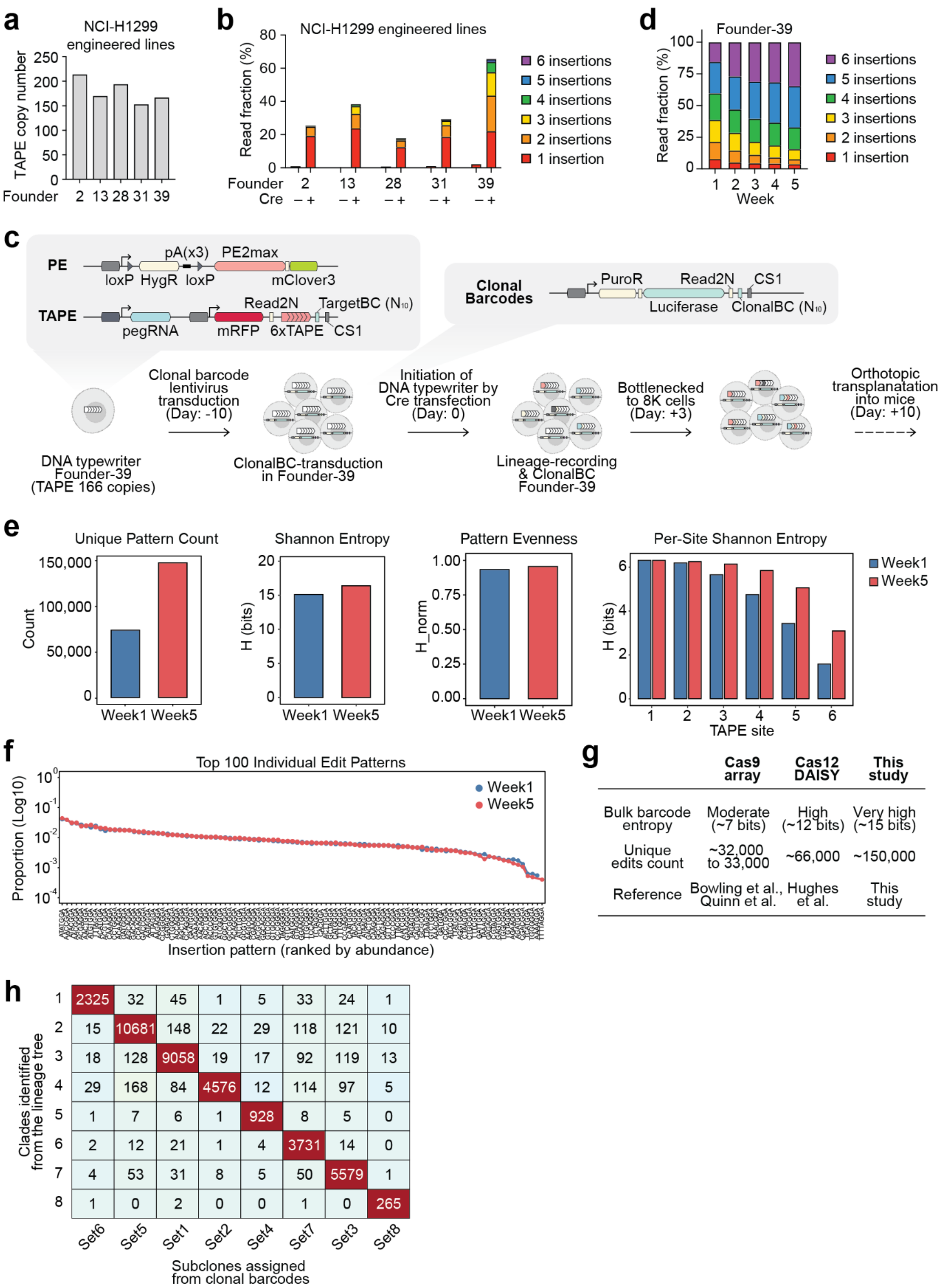


**Supplementary Figure 1. Engineering and benchmarking of the DNA Typewriter recording line.**
(a) Estimated TAPE copy number in each candidate NCI-H1299 line (five top ones among 48 screened shown), from the number of unique TargetBCs recovered by bulk amplicon sequencing.

bioRxiv preprint doi: https://doi.org/10.64898/2026.08.10.744013; this version posted August 12, 2026. The copyright holder for this preprint (which was not certified by peer review) is the author/funder, who has granted bioRxiv a license to display the preprint in perpetuity. It is made available under a CC-BY 4.0 International license.

(b) Recording efficiency and specificity in each candidate line without and with Cre transfection (Cre -/+), scored as the read fraction with 1 to 6 insertions.
(c) Construct schematics and workflow for generating clonally barcoded recording cells: the Cre-activatable Prime Editor (LSL-PEmax-P2A-mClover3), the 6xTAPE recorder (pegRNA-mRFP-6xTAPE-TargetBC), and the clonal-barcode construct (luciferase-ClonalBC-puromycin resistance), with the timeline (ClonalBC transduction day -11, Cre activation day 0, bottlenecking day 3, transplantation day 10).
(d) Recording by Founder-39 over the five-week window matching the in vivo experiment (day 17 to day 45), as the read fraction with 1 to 6 insertions per week.
(e) Recording diversity of Founder-39 at day 17 versus day 45 (weeks 1 and 5): unique pattern count, Shannon entropy, pattern evenness, and per-site Shannon entropy. Unique patterns increase and editing continues to reach the later TAPE sites while evenness stays high.
(f) Frequencies of the individual insertion sequences (NNNNGGA) at day 17 versus day 45, showing a stable insertion spectrum over time.
(g) Comparison of DNA Typewriter recording with Cas9-array and Cas12a (DAISY) lineage recorders by barcode size, bulk barcode entropy, and number of unique edits.
(h) Cross-tabulation of the subclone lineage-tree clades (rows) against the clonal-barcode sets (columns), with the number of cells in each clade-barcode pair. The near-diagonal structure shows that each clade corresponds to a single clonal-barcode set.

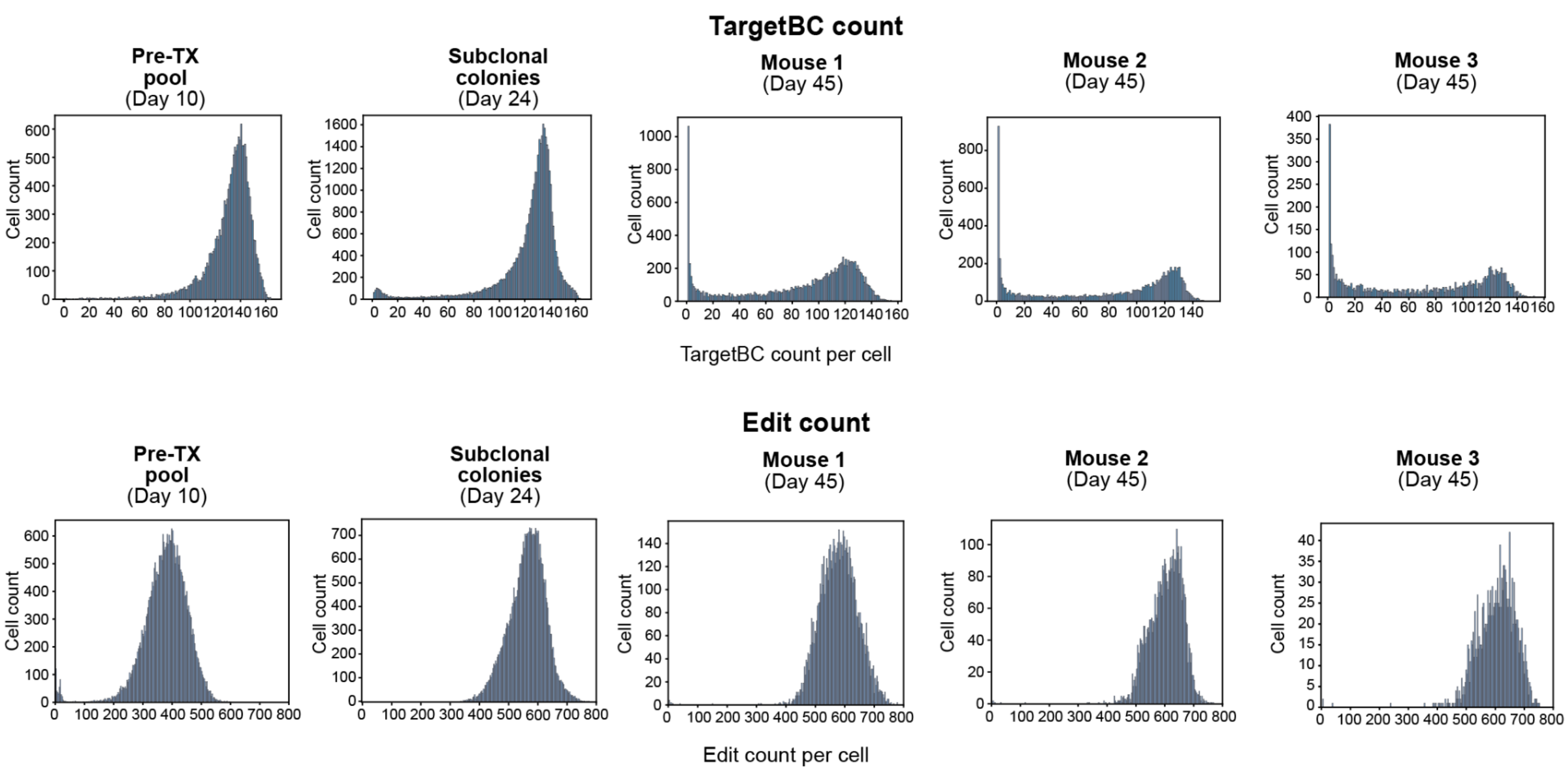


**Supplementary Figure 2. Per-cell recovery of lineage barcodes across samples.**
For each sample (the pre-transplantation pool, the in vitro subclonal colonies, and Mouse 1 to Mouse 3), distributions of (top) the number of TargetBCs recovered per cell, and (bottom) the number of TAPE insertions recovered per cell. In vitro cells recover most of the 166 TAPEs and several hundred edits per cell, and edit counts rise from the early pool to the week-5 in vivo samples.

bioRxiv preprint doi: https://doi.org/10.64898/2026.08.10.744013; this version posted August 12, 2026. The copyright holder for this preprint (which was not certified by peer review) is the author/funder, who has granted bioRxiv a license to display the preprint in perpetuity. It is made available under a CC-BY 4.0 International license.

bioRxiv preprint doi: https://doi.org/10.64898/2026.08.10.744013; this version posted August 12, 2026. The copyright holder for this preprint (which was not certified by peer review) is the author/funder, who has granted bioRxiv a license to display the preprint in perpetuity. It is made available under a CC-BY 4.0 International license.

chords link clones shared between the primary tumor and the metastatic sites, so the number of shared clones reports the clonality of each site.
(d) Global proliferation rank of clones (lambda or λ; rank 1 = fastest-growing). Point size scales with the clone's frequency in that condition. In vivo frequencies are computed from the per-cell clonal barcodes.
(e) Frequency-weighted mean lambda for each condition, against the mean of the fastest-growing 10% of clones (dashed line).

---

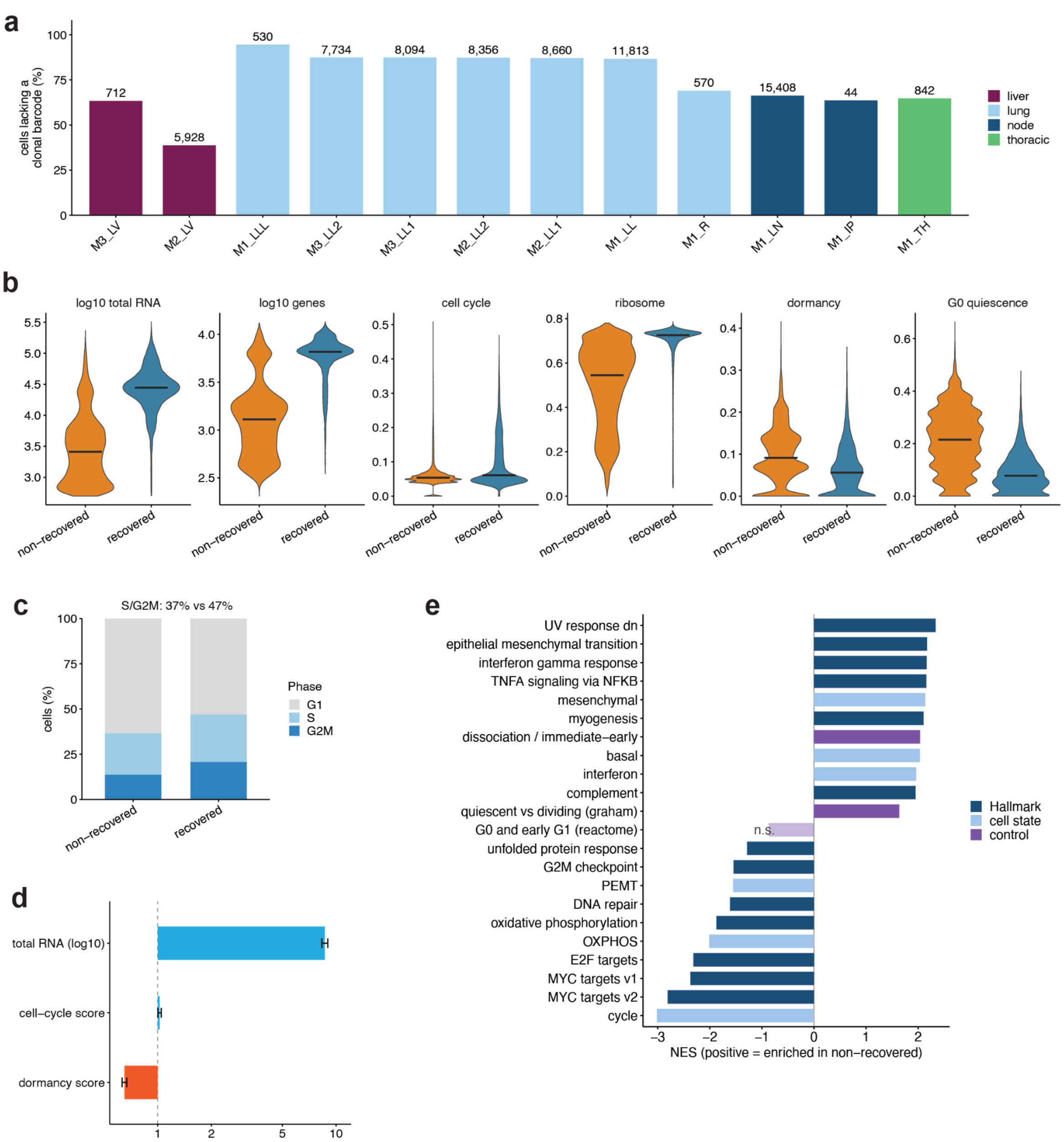

bioRxiv preprint doi: https://doi.org/10.64898/2026.08.10.744013; this version posted August 12, 2026. The copyright holder for this preprint (which was not certified by peer review) is the author/funder, who has granted bioRxiv a license to display the preprint in perpetuity. It is made available under a CC-BY 4.0 International license.

**Supplementary Figure 3. A pervasive low-recovery population is transcriptionally low-output and non-proliferative.**

(a) Fraction of transcriptomically defined tumor cells lacking a recovered clonal barcode, per sample (mouse and tissue; cell number above each bar), colored by tissue class. Non-recovery is lowest in the Mouse 2 liver metastasis (39%) and highest in the lung (87% to 95%), with the lymph nodes, thoracic mass, right lung, and the much smaller Mouse 3 liver metastasis intermediate (63% to 69%). The liver value is therefore driven by Mouse 2; the 712-cell Mouse 3 liver sits with the intermediate group.

(b) Recovered (blue) versus non-recovered (orange) cells for total RNA and detected genes (log10) and the cell-cycle, ribosome, dormancy, and G0-quiescence UCell scores. Non-recovered cells are low-output and low-ribosome. Per-cell rank-based scores are depressed by dropout in low-complexity cells, so the pseudobulk enrichment in (e) is the primary readout.

(c) Cell-cycle phase composition: non-recovered cells are less often in S or G2M (37% versus 47%).

(d) Logistic model of recovery (odds ratio per standard deviation, tissue-adjusted): recovery is dominated by transcriptional output (total RNA, odd ratio 8.6 per SD), while a higher dormancy score predicts lower recovery once output is adjusted for (odd ratio 0.65) and the cell-cycle score is flat (odd ratio 1.02).

(e) Module enrichment of the low-recovery population (primary characterization; same method as Figure 5b,c): fgsea on the depth-matched paired pseudobulk ranking against the Hallmark (dark) and pan-cancer cell-state (light) collections, with a dissociation/immediate-early set and two published quiescence references scored as pathways in the same run for direct comparison (purple; n.s. marks a control that is not enriched); NES > 0 = enriched in non-recovered. Proliferation (cell-cycle module, *MYC* and *E2F* targets, G2M), DNA repair and OXPHOS fall; Hallmark EMT, the mesenchymal module, interferon and inflammatory programs rise, while the narrower pEMT module falls. Of the two quiescence controls, the quiescent-versus-dividing reference rises (NES 1.64), but the mechanistic G0 and early-G1 pathway is not enriched (NES -0.87, adjusted P = 0.72), so the cell population is non-proliferative without showing a G0 entry program. The dissociation control also rises, which is why the claim rests on the falling axis. Abbreviations: NES, normalized enrichment score; UMI, unique molecular identifier.

bioRxiv preprint doi: https://doi.org/10.64898/2026.08.10.744013; this version posted August 12, 2026. The copyright holder for this preprint (which was not certified by peer review) is the author/funder, who has granted bioRxiv a license to display the preprint in perpetuity. It is made available under a CC-BY 4.0 International license.

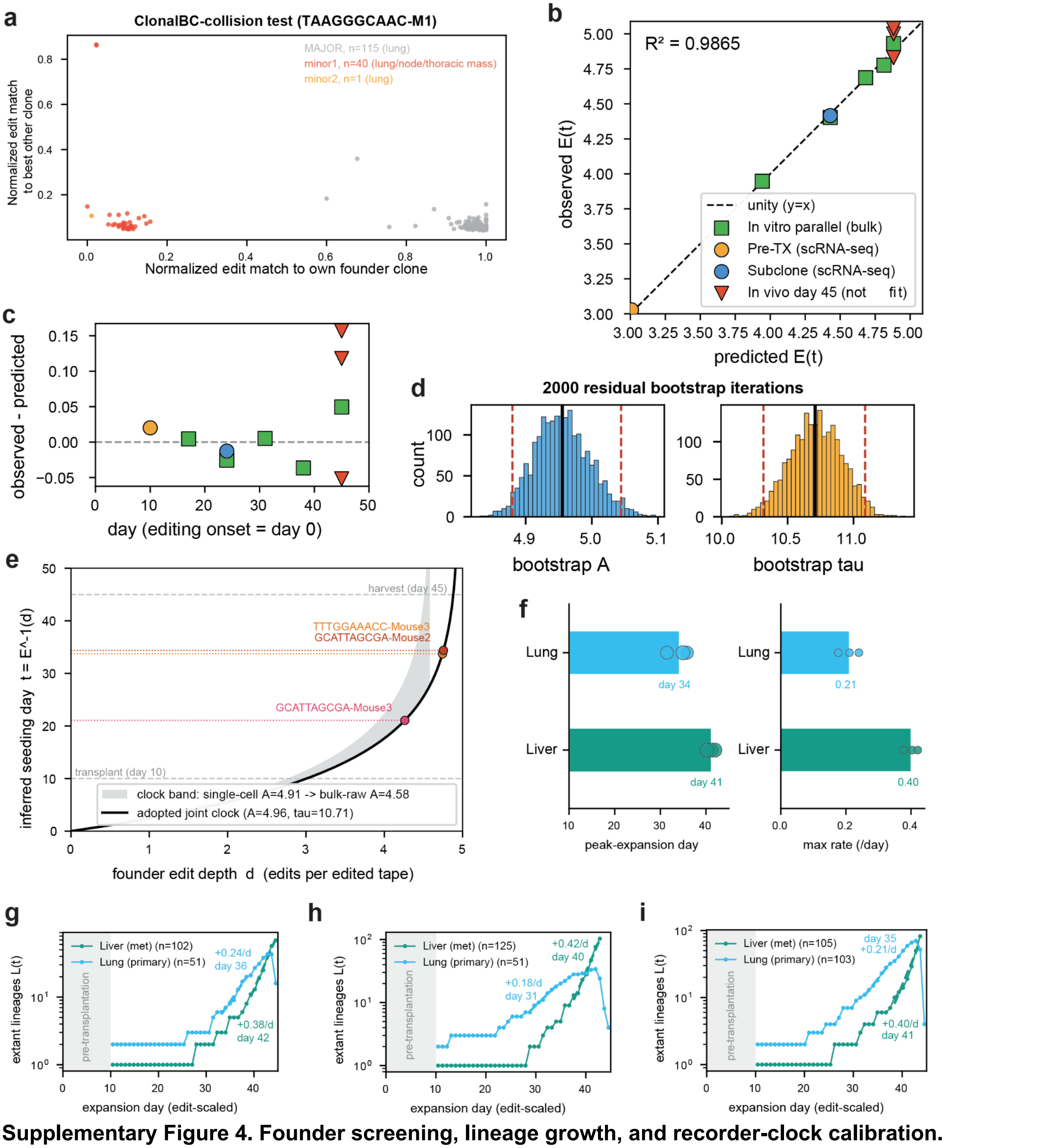


**Supplementary Figure 4. Founder screening, lineage growth, and recorder-clock calibration.**
(a) Founder and barcode-collision screen, which distinguishes a genuinely polyclonal barcode from a monoclonal seeding. The polyclonal example TAAGGGCAAC (Mouse 1) separates into a lung-restricted family (grey, 115 cells) and a distinct lymph-node and thoracic mass family (red, 40 cells) that share only the static clonal barcode, plus a single further cell assigned to neither (orange).

bioRxiv preprint doi: https://doi.org/10.64898/2026.08.10.744013; this version posted August 12, 2026. The copyright holder for this preprint (which was not certified by peer review) is the author/funder, who has granted bioRxiv a license to display the preprint in perpetuity. It is made available under a CC-BY 4.0 International license.

(b) Clock goodness-of-fit: observed versus predicted edit depth E(t) across the bulk in-vitro calibration series, the two single-cell anchors shown separately (Pre-TX at day 10 and Subclone at day 24), and the held-out in-vivo day-45 cells (R^2 = 0.99). Colours match the dataset series of Figure 3d.
(c) Calibration residuals (observed minus predicted E(t)) versus day, with the same series and colours as (g).
(d) Bootstrap distribution of the fitted clock parameters over 2,000 residual bootstrap iterations, with tau refitted holding the bulk series fixed. Solid black rule, the point estimate; dashed red rules, the 95% confidence interval. A = 4.9553 (95% CI 4.8799 to 5.0443) and tau = 10.7089 (95% CI 10.3167 to 11.0886), so the calibration is well-constrained.
(e) Dissemination-time sensitivity to the clock, confirming that the two near-saturation clones are robustly late.
(f) Per-tissue lineage growth. Left, the day of peak lineage expansion for each tissue on the edit-scaled tree, whose depth is anchored linearly from transplantation (day 10) to harvest (day 45); the lung primary precedes the liver metastasis in every clone, with no overlap (lung mean day 34.1, range 31 to 36; liver mean day 41.1, range 40 to 42). Right, the maximum instantaneous expansion rate per tissue (lung mean 0.21 per day, liver 0.40 per day). Both quantities are invariant to a constant sampling fraction, so the comparison is robust to the primary being under-sampled relative to the liver. The order, not the absolute rate, is reported, because the axis orders expansion rather than dating it and the near-saturation liver clade is compressed on the edit-scaled axis.
(g-i) Per-clone lineage-through-time curves underlying panel (f), one per dated clone. GCATTAGCGA-M2 in (g), GCATTAGCGA-M3 in (h), and TTTGGAAACC-M3 in (i).

bioRxiv preprint doi: https://doi.org/10.64898/2026.08.10.744013; this version posted August 12, 2026. The copyright holder for this preprint (which was not certified by peer review) is the author/funder, who has granted bioRxiv a license to display the preprint in perpetuity. It is made available under a CC-BY 4.0 International license.

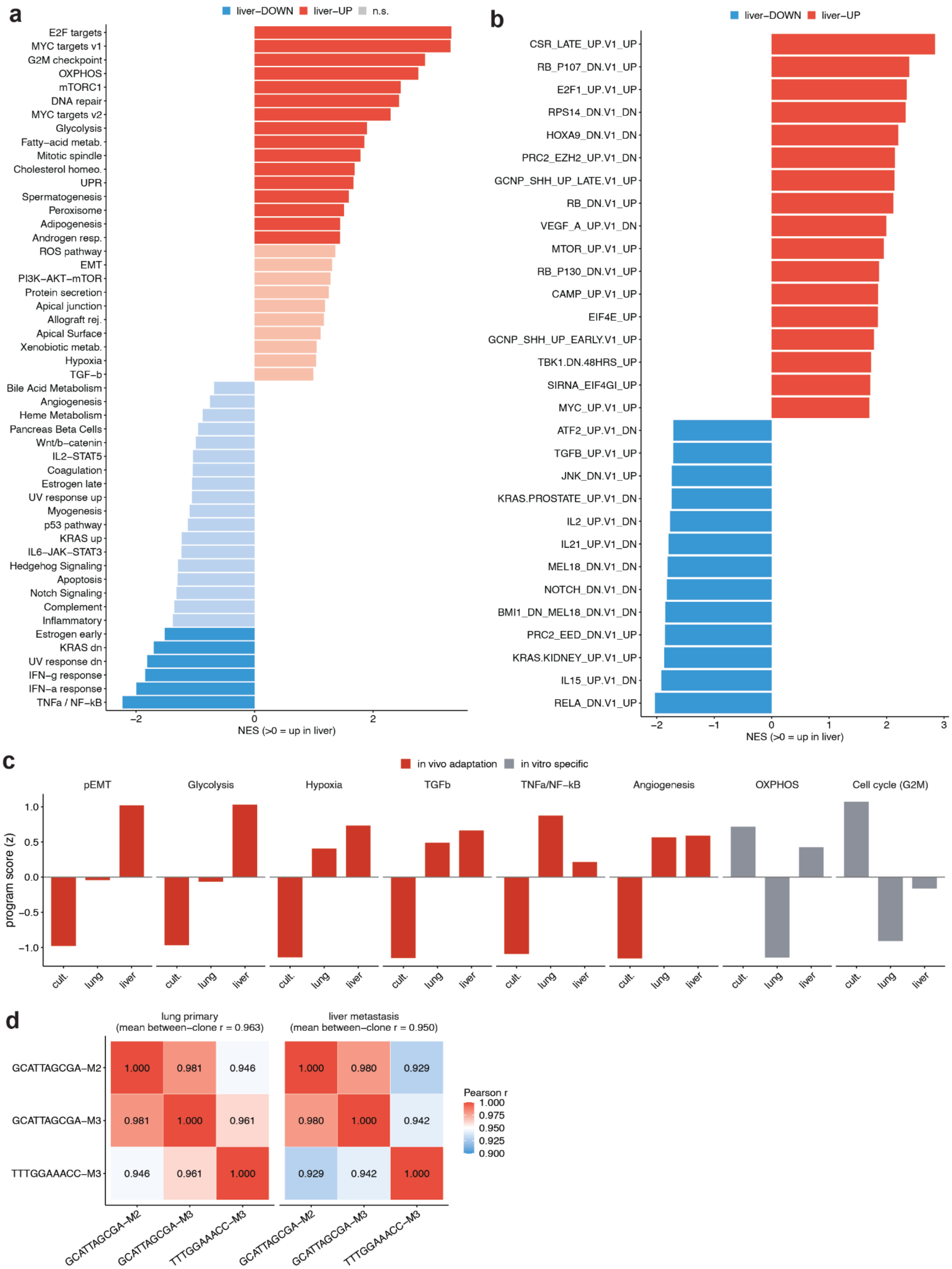


**Supplementary Figure 5. Extended liver-versus-lung enrichment, the three-environment trajectory, and between-clone similarity.**

(a) All 50 Hallmark gene sets ranked by normalized enrichment score (NES) for the liver-versus-lung contrast, from the pooled paired pseudobulk ranking (fgsea; NES > 0 = enriched in the liver). Bars are colored by direction and shaded by significance; 22 of the 50 sets reach a 5% false-discovery rate.

bioRxiv preprint doi: https://doi.org/10.64898/2026.08.10.744013; this version posted August 12, 2026. The copyright holder for this preprint (which was not certified by peer review) is the author/funder, who has granted bioRxiv a license to display the preprint in perpetuity. It is made available under a CC-BY 4.0 International license.

(b) The 30 most extreme oncogenic (C6) signatures[26] of the 189 tested, ranked by NES for the same contrast. The RB-loss and E2F-activation signatures corroborate the proliferative axis of **Figure 5b**.
(c) Culture to lung to liver program trajectory, one small multiple per program. Bars are the mean program score (UCell[31]) averaged over the three genotype-matched clones and z-scored across the three environments, and are colored by trajectory shape: red, programs induced in vivo relative to culture, including the liver-adaptation programs; grey, programs highest in culture that fall in the lung and recover only partly in the liver.
(d) Between-clone transcriptome similarity by environment (Pearson correlation over 2,000 highly variable genes), for the lung primary (left) and the liver metastasis (right). Clones remain transcriptionally distinct in both tissues, and the liver metastases are no more similar to one another than the primaries are (mean between-clone r = 0.963 in the lung, 0.950 in the liver).

bioRxiv preprint doi: https://doi.org/10.64898/2026.08.10.744013; this version posted August 12, 2026. The copyright holder for this preprint (which was not certified by peer review) is the author/funder, who has granted bioRxiv a license to display the preprint in perpetuity. It is made available under a CC-BY 4.0 International license.

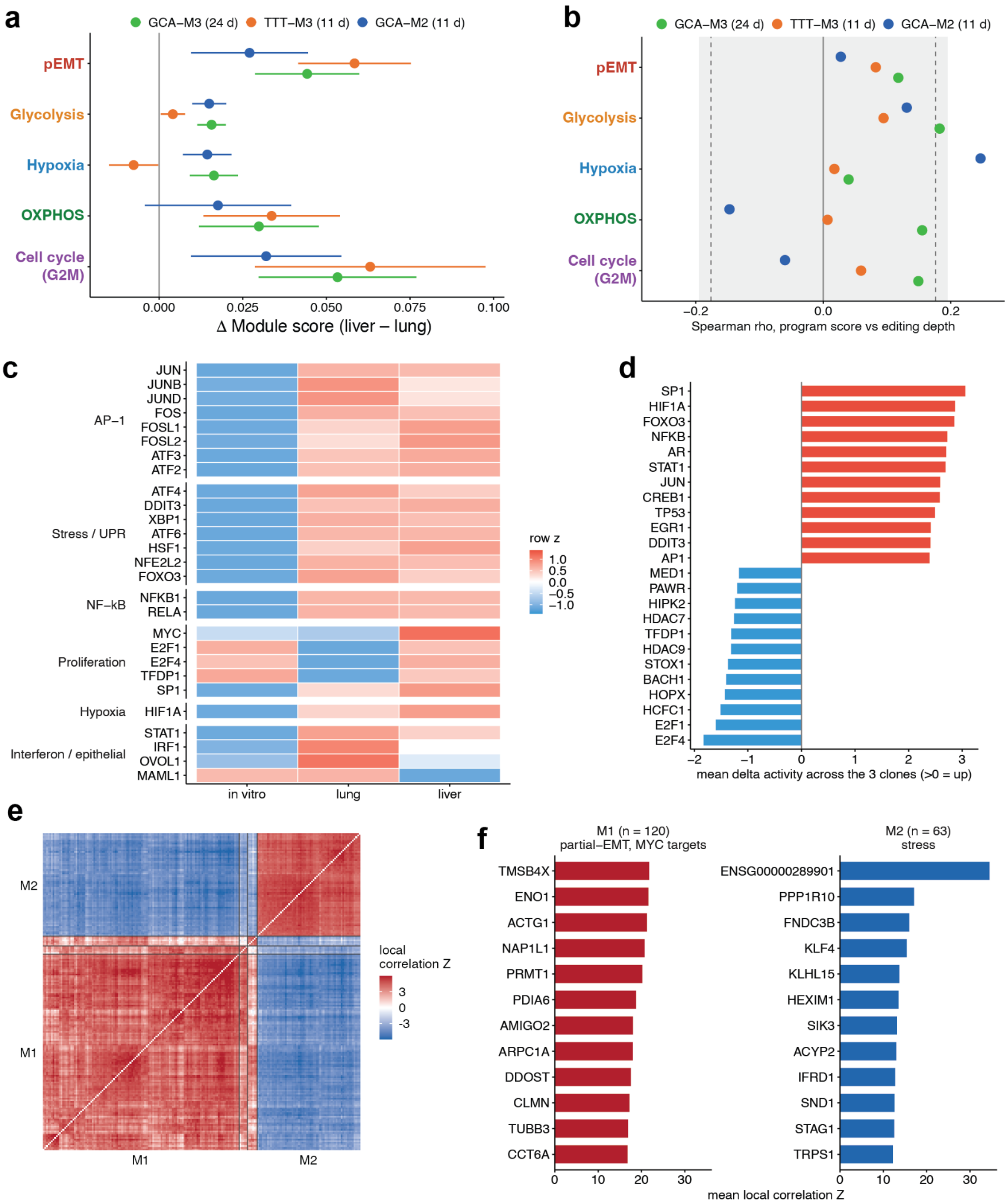


**Supplementary Figure 6. Residence time and the transcription-factor regulators of the liver-adaptation program.**

(a) Liver-minus-lung difference in each of the five liver-adaptation programs, one series per clone, with 95% confidence intervals from the per-cell scores. Clones are labelled with their residence time in the liver at harvest (harvest day minus the dissemination day of **Figure 3**). The longest-resident metastasis, at 24 days, is not the most adapted in any program, and the two seeded about 11 days before harvest reach the same or larger differences, so the program is installed rapidly rather than deepening over this window.

(b) Internal control for (a). Within each liver metastasis, the per-cell Spearman correlation between each program's score and that cell's TAPE-recorder editing depth (332 liver cells over three clones). The shaded

bioRxiv preprint doi: https://doi.org/10.64898/2026.08.10.744013; this version posted August 12, 2026. The copyright holder for this preprint (which was not certified by peer review) is the author/funder, who has granted bioRxiv a license to display the preprint in perpetuity. It is made available under a CC-BY 4.0 International license.

band is the two-sided 5% significance range, $1.96/\sqrt{(n - 1)}$, drawn as a range because n differs between clones; the dashed lines mark its narrowest extent.

(c) Curated regulon activity across the three environments (row z-score), grouped by family (AP-1, stress and unfolded-protein response, NF-kB, proliferation, hypoxia, interferon and epithelial). Cell means are averaged within each clone first and then across clones, to avoid the domination of the liver column by the single 3,387-cell clone (ATGATCATAT-M2).

(d) Data-driven regulon activity of the culture-to-lung entry step (decoupleR[27] over the CollecTRI network[28]), showing the twelve transcription factors rising and the twelve falling most by across-clone mean activity.

(e) Cross-clone consensus of the lineage-informed co-expression structure in the three liver metastases. Hotspot was run per clone with that clone's edit-scaled single-cell lineage tree as the cell-to-cell metric; each clone's local correlation matrix was then averaged across clones and clustered, over the 194 genes recurrently autocorrelated among them. Blocks on the diagonal are the four consensus modules, of which the two large ones are labelled (M1, 120 genes; M2, 63). Module numbering is independent of the in-vitro consensus of **Figure 6a**. Colour is the averaged local correlation Z, clipped at its 99th percentile; the diagonal is blank because self-correlations are excluded.

(f) The twelve strongest genes of each of the two large consensus modules of (e), ranked by mean local correlation Z, with the programs that module is enriched for shown above it (one-sided hypergeometric over the 194 genes entering the consensus). The two anti-correlated blocks of (e) are an adaptation and biosynthesis module set against a stress module (7 of 7 Barkley stress genes, $P = 3 \times 10^{-4}$). The adaptation module is enriched for partial-EMT (14 of 14 set members present, $P = 9 \times 10^{-4}$) and *MYC* targets (19 of 22, $P = 0.008$), and carries the cytoskeletal and glycolytic genes *TMSB4X*, *ACTG1* and *ENO1*. The strongest gene of the stress module, *ENSG00000289901*, is a 218-base unnamed lncRNA on chromosome 13 with no assigned symbol; it is recovered independently in all three metastases but is not in the heritable set of **Figure 6** and is null in the pre-transplant outcome-prediction screen in **Figure 6**.

bioRxiv preprint doi: https://doi.org/10.64898/2026.08.10.744013; this version posted August 12, 2026. The copyright holder for this preprint (which was not certified by peer review) is the author/funder, who has granted bioRxiv a license to display the preprint in perpetuity. It is made available under a CC-BY 4.0 International license.

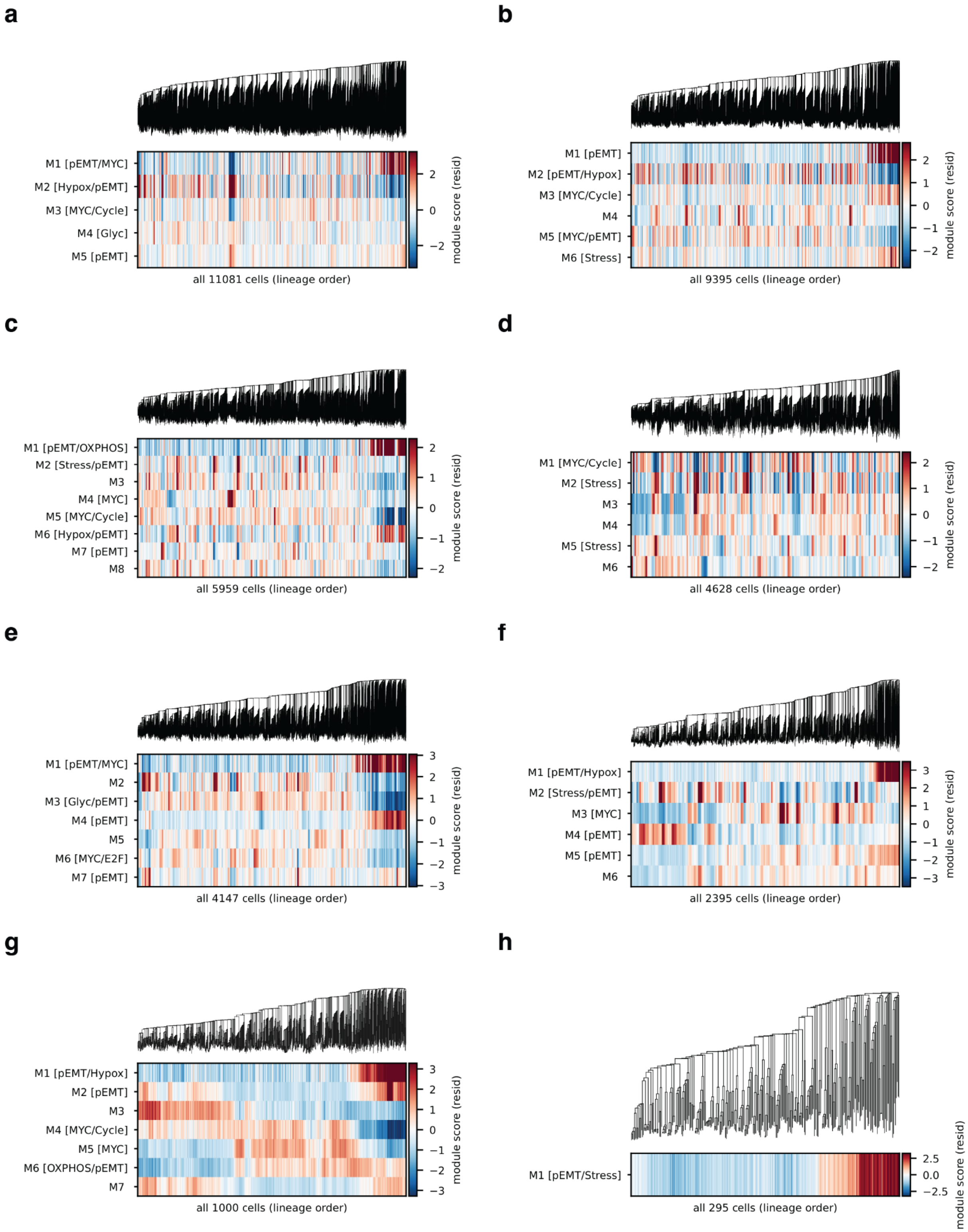


**Supplementary Figure 7. Lineage heritability in each of the eight monoclonal pre-transplant colonies.** (a–h) One panel per colony, ordered by cell number: the full-depth Cassiopeia lineage tree above a heatmap of per-cell heritable-module scores in lineage order, residualized on mitochondrial fraction and log total counts so that a block along the tree is not a depth or quality gradient. Colonies contain 295 to 11,081 cells and yield 86 to 2,283 lineage-autocorrelated genes (Hotspot FDR < 0.05). Module numbering and annotation are assigned independently within each colony and do not correspond between panels, nor to the consensus modules of

bioRxiv preprint doi: https://doi.org/10.64898/2026.08.10.744013; this version posted August 12, 2026. The copyright holder for this preprint (which was not certified by peer review) is the author/funder, who has granted bioRxiv a license to display the preprint in perpetuity. It is made available under a CC-BY 4.0 International license.

**Figure 6a**. The smallest colony (h, 295 cells) is power-limited and resolves a single module, which is partial-EMT annotated. Panel (g) is the colony shown in **Figure 6a**.

---

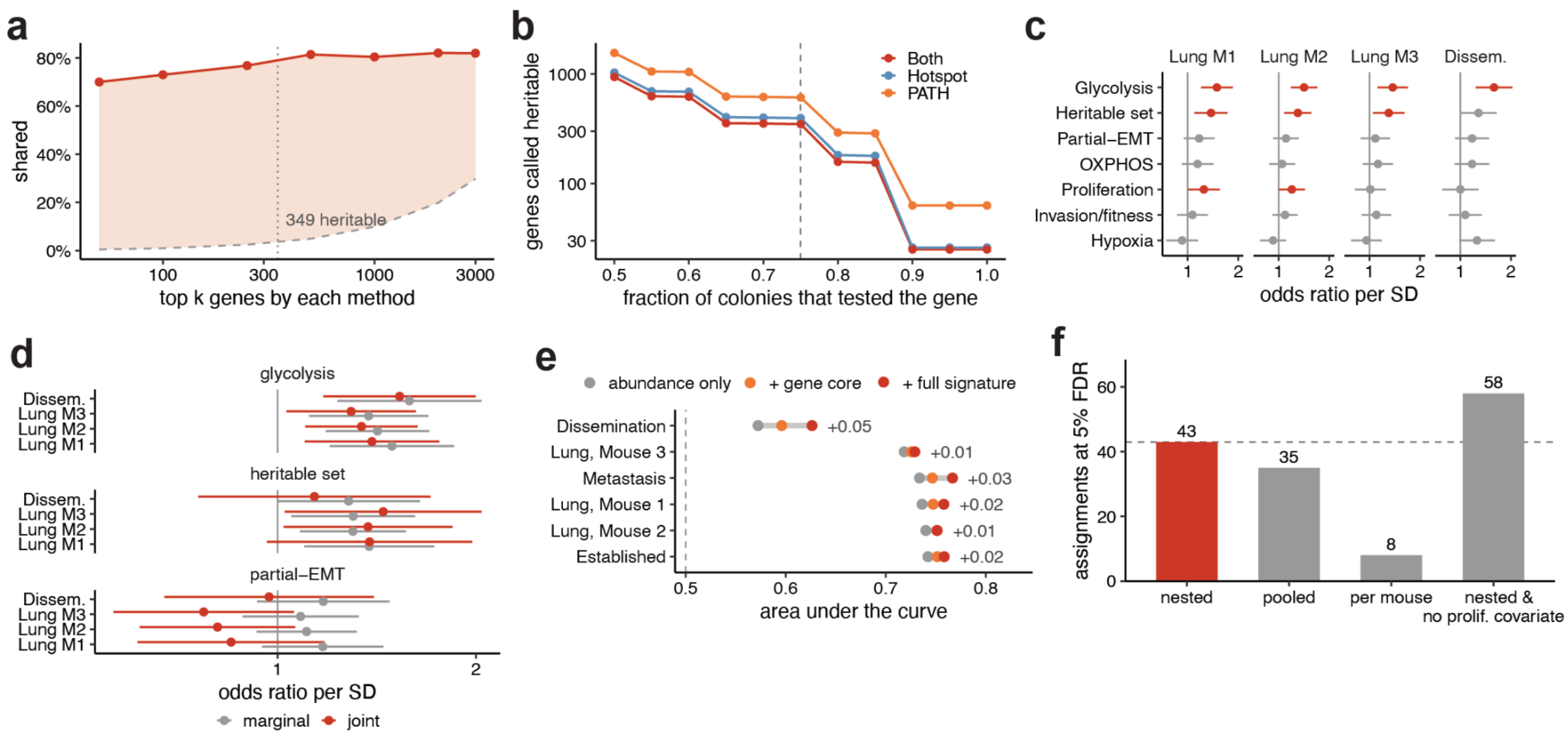


**Supplementary Figure 8. Robustness of the heritability, prediction and gene-scan results of Figure 6.**
(a) Overlap of the top k genes ranked by mean Hotspot Z and by mean PATH Z, over the 10,078 genes scored by both. Red, observed; dashed grey, chance overlap at that k; shading, the excess over chance. Dotted line, the 349-gene heritable set.
(b) Number of genes called heritable against the recurrence fraction, for each method and their intersection, requiring at least four colonies tested throughout. Dashed line, the 75% used in the main figures.
(c) All signatures fitted alone and adjusted for pool abundance, across the three primary-lung outcomes and dissemination. Red, $P < 0.05$. Proliferation reaches nominal significance in Mouse 1 and Mouse 2 but not Mouse 3 and not for dissemination, with both confidence intervals bounded at one.
(d) Odds ratio per standard deviation for the three competence signatures, each adjusted for pool abundance. Grey, each fitted alone; red, all three fitted together. Glycolysis and the outcome-independent heritable set both retain their effect on colonization under joint adjustment. Partial-EMT inverts below one in every contrast.
(e) Area under the curve for each outcome from models containing pool abundance alone (grey), abundance plus a compact gene core (14 heritable glycolysis gene of *CITED2, ENO1, FUT8, HDLBP, LDHA, MIF, P4HA1, PAM, PGK1, PKM, PPIA, SOX9, TPI1, TXN*; orange), and abundance plus the full signature (191 MSigDB glycolysis set[26]; red); the segment spans abundance alone to the full model and the number is the gain. Each mouse's primary lung is tested separately. "Established" is recovery anywhere versus never. “Metastasis” includes any secondary sites excluding left lung, and "Dissemination" compares metastasis with lung-only clones, excluding clones never recovered.
(f) Gene-outcome assignments passing 5% FDR under four outcome-labelling schemes; red, the nested scheme used in the main figure (dissemination nested in the established clones), with the dashed line at its count of 43.

bioRxiv preprint doi: https://doi.org/10.64898/2026.08.10.744013; this version posted August 12, 2026. The copyright holder for this preprint (which was not certified by peer review) is the author/funder, who has granted bioRxiv a license to display the preprint in perpetuity. It is made available under a CC-BY 4.0 International license.

**Supplementary Note 1. Low-TAPE/TargetBC recovery in vivo cell population.**
All three mice and both the primary lung and the liver metastases were analyzed together (68,691 single cells from twelve samples; **Methods**). Low recovery was pervasive: 77% of transcriptomically defined tumor cells yielded no clonal barcode. The fraction varied by site along a reproducible gradient (**Supplementary Figure 3a**), highest in the lung primary (86 to 95%) and the peripheral thoracic sites (lymph node, thoracic mass, and intraperitoneal nodes, 64 to 67%) and lowest in the liver metastasis (39% in Mouse 2, 64% in Mouse 3).

Relative to recovered cells they had roughly 10-fold lower total RNA, about 5-fold fewer detected genes, markedly lower ribosomal and translational gene expression, and a lower cycling fraction (34% versus 46% in S or G2M) (**Supplementary Figure 3b,c**). Because recovery of the clonal barcode and DNA Tape is an amplicon measurement that scales with transcriptional output, recovery is itself largely a readout of that output: in a logistic model the odds of recovery rose roughly eight-fold per standard deviation of total RNA (**Supplementary Figure 3d**).

To characterize the state without presupposing a marker set, we applied the same module-enrichment approach used for the liver adaptation, testing paired pseudobulk differential expression (recovered versus non-recovered within each sample) against the Hallmark and pan-cancer cell-state collections (**Supplementary Figure 3e**; **Methods**). The dominant and most consistent feature was a coordinate shutdown of proliferation and biosynthesis in the low-recovery cells: *MYC* targets (normalized enrichment score or NES = -3.0), E2F targets (-2.2), the G2M checkpoint (-1.5), DNA repair (-1.6) and OXPHOS (-1.7) were all depleted, and the pan-cancer cell-cycle module was the most depleted module in every contrast (NES = -3.0 depth-matched, -2.9 in the lung, -3.5 in the liver). A published quiescent-versus-dividing signature was independently enriched (NES = 1.8, FDR = $1 \times 10^{-4}$).
